# Barriers and facilitators to implementation of developmental screening and early intervention in Canadian organizations following completion of a training and coaching model: a thematic analysis

**DOI:** 10.3389/frhs.2023.1160217

**Published:** 2023-06-23

**Authors:** Karys Peterson-Katz, Caitlin Piccone, Nicole Tuzi, Chaya Kulkarni, James N. Reynolds

**Affiliations:** ^1^ Centre for Neuroscience Studies, Queen’s University, Kingston, ON, Canada; ^2^ School of Rehabilitation Therapy, Queen’s University, Kingston, ON, Canada; ^3^ Infant and Early Mental Health Promotion, The Hospital for Sick Children, Toronto, ON, Canada

**Keywords:** early intervention, child development, implementation, ages and stages, infant mental health, developmental delay, Canadian context

## Abstract

**Introduction:**

Developmental delay affects approximately 1 in 4 children under 6 years old. Developmental delay can be detected using validated developmental screening tools, such as the Ages and Stages Questionnaires. Following developmental screening, early intervention can occur to address and support any developmental areas of concern. Frontline practitioners and supervisors must be trained and coached to organizationally implement developmental screening tools and early intervention practice. No prior work has qualitatively investigated the barriers and facilitators to implementing developmental screening and early intervention in Canadian organizations from the perspectives of practitioners and supervisors who have completed a specialized training and coaching model.

**Methods and Results:**

Following semi-structured interviews with frontline practitioners and supervisors, thematic analysis identified four themes: cohesive networks support implementation efforts, implementation success is dependent on shared perspectives, established organizational policies increase implementation opportunities, and COVID-19 guidelines create organizational challenges. Each theme encompasses sub-themes that describe implementation facilitators: strong implementation context, multi-level multi-sectoral collaborative partnerships, adequate and collective awareness, knowledge, and confidence, consistent and critical conversations, clear protocols and procedures, and accessibility to information, tools, and best practice guidelines.

**Discussion:**

The outlined barriers and facilitators fill a gap in implementation literature by informing a framework for organization-level implementation of developmental screening and early intervention following training and coaching.

## 1. Introduction

In Canada, approximately 1 in 4 children are reaching school age with measurable vulnerabilities in multiple areas of development, including overall wellbeing, cognitive development, emotional maturity, communication skills, and general knowledge (1). The first 5 years of life are a critical period when programs that support early childhood development can have an impact on lifelong trajectories of physical and mental health, academic success, employment stability, and financial literacy (2, 3).

During this critical early period, young children are highly responsive to external influences that can shape their developmental trajectory. External influences can include programs that support childhood development, otherwise known as early intervention. Evidence shows that early intervention, particularly within the child's first year of life, predicts more positive lifelong outcomes, and can positively affect physical skills, cognition, social-emotional well-being, academic success, social independence, and caregiver-child interactions (4–6).

While early intervention has key benefits to development, not all children are able to receive these services. Eligibility for early intervention services is contingent on several factors, including availability and accessibility of services. Accessing early intervention services often depends on developmental screening to determine which of the child's abilities may benefit from additional support, as well as the level of support required. Developmental screening tools are generally brief and evaluate a child's risk for developmental delay in one or more domains (7). Although screening tools are not diagnostic (8), they are still beneficial because any trained service provider can identify developmental vulnerabilities in a time- and resource-efficient manner (9).

Multiple factors influence and perpetuate developmental vulnerabilities. For example, socioeconomic status, social and physical environments, trauma, and adverse experiences are all determinants of long-term health outcomes. Notably, it has been found that adverse childhood experiences have a graded, continuing relationship with overall lifetime well-being that begins in the early years (10).

However, while there are various risk factors for negative developmental trajectories, there are also protective factors for the development of disadvantaged children as well, such as strong child-caregiver attachment (11), and high-quality childcare (4, 12). High-quality childcare depends on competent, trained childcare practitioners that are able to recognize and address early developmental needs and areas of developmental concern. Recognizing and addressing need can be accomplished by administering developmental screening and providing early intervention services. Well-trained early childhood practitioners have been found to have positive impact on child outcomes, such as school readiness (13), academic performance (2), and enriched cognitive and social-emotional development (14).

Practitioners who work with young children and want to provide high-quality childcare can benefit from training and coaching related to early child development. Early childhood service provider training has been shown to increase awareness and knowledge related to infant and early mental health and development (15), as well as confidence and understanding (16). However, while training is beneficial to shift awareness, knowledge, confidence, and understanding regarding early developmental need, training alone may not be sufficient for practitioners to commit consistently to screening and early intervention practices (17, 18).

Coaching following training has been demonstrated to be an essential aspect of professional development to ensure practitioners embed new skills into routine practice (19). Coaching allows for practitioners to be assisted in the learning process, as coaches can offer support and feedback- key factors to guided behaviour change (20, 21). In fact, practitioners who only received training were found to provide lower quality childcare practice (19, 22), have slower skill acquisition (23), and lower likelihood of successful skill implementation that is sustained long-term (20, 23), than those who also took part in professional development coaching.

Although there is substantial evidence that early intervention supports typical developmental trajectories for children in different contexts (24–26) and training and coaching on early intervention practice supports implementation (27), there is inconsistent implementation of developmental screening and early intervention in Canadian organizations that provide care to children under the age of 6 (28, 29).

There are two key benefits to identifying and evaluating barriers and facilitators to implementation of early intervention tools in community-based organizations that work directly with children under the age of 6 years. First, the information gained can be used to promote learning and capacity-building to address and overcome identified challenges (30, 31). Second, understanding the factors that enable successful implementation of screening tools and early intervention practice can reinforce the current training and coaching model, or provide insight to effectively modify this model (32). Understanding the barriers and facilitators to implementation of new practices at the organizational level can provide insight into the steps necessary to bolster programs that support early identification and intervention in young Canadian children.

## 2. Methods

### 2.1. Infant and early mental health promotion training and coaching

Infant and Early Mental Health Promotion (IEMHP), a program of the Hospital for Sick Children in Toronto, Ontario, partnered with the Knowledge Institute for Child and Youth Mental Health and Addictions to institute a Care Pathways pilot project. At the time of data collection, this project partnered with three Ontario communities to: (1) support families by working with service providers to increase communication and transparency between sectors, (2) train practitioners on developmental screening initiatives, (3) identify gaps in organizational policies, and (4) produce data on early mental health prevalence and program efficacy.

Between October 2020 and September 2021, 458 individuals completed the training and coaching model. The training was divided into two separate streams: Stream 1 for organization supervisors, and Stream 2 for frontline practitioners. The participants were categorized into one of 7 identified sectors based on their primary professional affiliation: (1) Early Learning and Care (e.g., Drop-in Centres), (2) Education (e.g., Kindergarten), (3) Child Mental Health, (4) Public Health, (5) Child Welfare, (6) Primary Health Care, and (7) Rehabilitation.

The IEMHP training and coaching model evaluated in this study consisted of the following, completed in the order listed: (1) a 5-h asynchronous introductory webinar series on infant and early mental health, completed at the individual user's pace, (2) a 2-h asynchronous virtual introduction to the Ages and Stages Questionnaires (ASQs), completed at the individual user's pace, (3) a 1.5-h online synchronous coaching session with an IEMHP group facilitator and up to 10 trainees from various community organizations, focused on use and application of the ASQs, (4) a 1.5-h asynchronous introductory webinar series on creating and implementing Developmental Support Plans (DSPs), completed at the individual user's pace, (5) a 1.5-h virtual synchronous coaching session with an IEMHP facilitator on use and application of the DSP, (6) five 1.5 h virtual coaching sessions allotted for each community organization to attend with an IEMHP facilitator to answer questions, provide clarity, and further coach the participants in the understanding of infant and early mental health, developmental screening, and early intervention.

### 2.2. Implementation study

To provide impactful training and coaching on infant and early mental health (IEMH), as well as early intervention practice, it is essential to gain insight into practitioner and supervisor experiences of: (1) learning through a training and coaching model and, (2) implementing early intervention practice. The objective of this qualitative study was to better understand the barriers and facilitators of implementing the Ages and Stages Questionnaire-3 (ASQ:3), Ages and Stages Questionnaire Social-Emotional:2 (ASQ:SE-2), henceforth referred to as “ASQs”, and Developmental Support Plans (DSPs), following completion of the training and coaching model on IEMH and early intervention practice described above. The present study focused on the experiences of practitioners in Ontario-based organizations working directly with children under the age of 6, as well as the experiences of the organization supervisors.

### 2.3. Study design

The Promoting Action on Research Implementation in Health Services (PARIHS) framework was used to inform this study (33–35). The PARIHS framework emphasizes the dynamic relationship between three essential implementation elements: evidence, context, and facilitation, including their respective sub-elements (34, 36). Successful implementation is reported as equally dependent on the quality of evidence, the context in which the evidence is introduced, the skill of the facilitators involved in implementation, and the method by which it is implemented (37).

The PARIHS framework is flexible and allows users to only include elements and sub-elements that address their target concerns, questions, and objectives (38, 39). This study held a main focus on the element of “context”, and a minor focus on the element of “facilitation”, as well as their respective sub-elements.

The PARIHS framework element of “context” and “facilitation”, and the affiliated sub-elements, guided the creation of the semi-structured interview guide, as well as data coding and interpretation. The primary researcher conducted semi-structured interviews to collect information on the barriers and facilitators to implementation of early intervention in child-centered organizations. Interviews were conducted with both supervisors and practitioners who contributed to implementation within their organizations.

### 2.4. Recruitment

Participants were recruited from the cohorts of practitioners and supervisors who had completed IEMHP's training and coaching model between October 2020 and September 2021. A mass e-mail invitation was sent out by IEMHP to participants using CVENT, a virtual event management and participant engagement tool. Participants who accepted the invitation to take part in the study had one-on-one virtual semi-structured interviews with the primary researcher (KP-K). See Table 1 for Participant Demographics.

**Table 1:** Participant demographics^a^.

Characteristics	Practitioners		Supervisors		Full sample	
	*n*	%	*n*	%	*n*	%
Gender						
Woman	7	100	4	100	11	100
Man	0	0	0	0	0	0
Other	0	0	0	0	0	0
Age						
18–24	0	0	0	0	0	0
25–34	0	0	0	0	0	0
35–44	3	43	1	25	4	36
45–54	2	29	1	25	3	27
55–64	1	14	1	25	2	18
65–74	0	0	1	25	1	9
Over 75						
Ethnicity/Race						
White	7	100	4	100	11	100
Black	0	0	0	0	0	0
Indigenous	0	0	0	0	0	0
East/Southeast	0	0	0	0	0	0
Asian	0	0	0	0	0	0
South Asian	0	0	0	0	0	0
Latino	0	0	0	0	0	0
Middle Eastern	0	0	0	0	0	0
Other						
Highest educational level						
High school diploma or equivalent	0	0	0	0	0	0
Some college but no diploma	0	0	0	0	0	0
College diploma or certificate	0	0	1	25	1	9
Bachelor's degree	4	57	2	50	6	55
Graduate degree	2	29	1	25	3	27
Sector						
Child Welfare	2	29	2	50	4	36
Early Learning and Care (ELC)	1	14	0	0	1	9
Child Mental Health	1	14	0	0	1	9
Primary Health Care	0	0	1	25	1	9
Public Health	2	29	1	25	3	27
Education	1	14	0	0	1	9
Years in sector						
<1 year	0	0	0	0	0	0
1–4 years	0	0	0	0	0	0
5–9 years	0	0	1	25	1	9
10–14 years	3	43	0	0	3	27
15–19 years	1	14	1	25	2	18
20+ years	2	29	2	50	4	36

^a^ One participant declined to disclose demographic information related to age, highest educational level, and years in the sector.

### 2.5. Procedure

All procedures were reviewed and approved by the Queen's University Health Sciences Research Ethics Board. Participants were provided with a letter of information (LOI) and consent form at least 1 day prior to the one-on-one interview to ensure they had adequate time to review the materials. At the beginning of the one-on-one meeting, prior to the commencement of the interview, the interviewer confirmed the participant had read and understood the LOI and consent form and obtained informed verbal consent to take part in the study, including publication and presentation of deidentified quotes. The interviews were recorded to enable verbatim transcription. At the end of the one-on-one interview, the participants were asked if they had any questions, if there was any clarification needed, or if there was any more information they would like to share. Participants were also given the option to receive a verbatim transcript following completion of the interview and transcription process. Each interview ranged from 45 to 60 min.

### 2.6. Data analysis

The primary researcher employed a Codebook thematic analysis approach (40–42) to engage with the data, identify themes, and encompass the barriers and facilitators of implementation that the practitioners and supervisors experienced. The initial Codebook was guided by the PARIHS framework and the research question and included: Barriers, Facilitators, Training, Coaching, Context, Leadership, and Facilitation. Participants were given pseudonyms to ensure anonymity, and the recorded interviews were transcribed verbatim for further analysis.

The primary researcher first read through each transcript fully, without coding or note-taking. The primary researcher then re-read each transcript sequentially, at this point beginning to code using the initial Codebook, take initial notes, and reflexive journaling. Reflexivity in qualitative research is a process of constantly and critically self-examining internal judgements, biases, and positionality in order to acknowledge that the researcher's subjectivity and personal experiences are inseparable from the data interpretations (43).

The initial notes focused on the tone of the participant's responses, (e.g., whether an experience appeared to be associated to positive or negative feelings), as well as perceived participant perspective. The reflexive journaling included the researcher's coding strategy, as well as initial interpretations based on the researcher's thought process throughout review and analysis of the transcripts.

Through integration with the data, the primary researcher identified that the initial Codebook was not sufficiently capturing the concepts discussed by all participants. At this stage, the primary researcher inductively coded using NVIVO 12 [for an in-depth resource review, see Dhakal (44)]. Following initial coding, the primary researcher noted initial themes, organizing codes into rudimentary themes. The themes were then reviewed by the primary researcher and re-organized. Theme re-organization continued until the primary researcher felt themes were clearly and comprehensively defined. A secondary researcher (CP) independently read through 3 of the transcripts and developed initial notes. The secondary researcher's initial notes focused on noticeable patterns. Following this step, the secondary researcher re-read the same three transcripts and generated a first draft of themes. Next, the secondary researcher coded the three initial transcripts in NVIVO 12, before coding the rest of the transcripts. The secondary researcher refined the themes throughout the process. The secondary researcher modified their “Codebook” to include initial codes, sub-codes, and descriptions for each code. At this stage, the primary researcher and secondary researcher met to discuss and compare codes and themes. During the meeting, the two researchers modified their Codebooks to reflect the shared understanding and interpretations that emerged throughout the discussion. Following the discussion, the primary researcher incorporated the elaborated understandings and interpretations when re-coding and re-interpreting the data. In this fashion, themes seen as “shallow” were discarded or refined, and substantial, meaningful themes were developed in their place.

Once themes were deemed appropriately meaningful, the primary researcher contacted the participants to request a follow-up meeting to discuss the synthesis of the analyzed data in order to enhance the trustworthiness of the findings, otherwise known as “member checking”. Member checking is a method of rigor to promote the accurate representation of participant perspectives and reduce researcher bias (45). Seven of the eleven participants agreed to a follow-up meeting with the primary researcher. One participant cancelled their meeting with the researcher and did not reschedule to a later date. The primary researcher thus met with the 6 participants who agreed to a follow-up discussion. Each meeting lasted approximately 60 min. Reflexivity occurred at this step as well, through mutual collaboration in a co-operative dialogue between researcher and participants (46). The primary researcher walked through all themes with each individual participant, taking notes on all clarification, elaboration, and feedback. Themes were discussed until the participants felt satisfied with the interpretations, at which point the interviews would conclude.

## 3. Results

### 3.1. Thematic analysis

A combination of deductive and inductive coding in a “Codebook” thematic analysis approach revealed 4 themes. Three themes had two sub-themes each. One theme did not contain sub-themes. In-text quotes from participants include their pseudonym, sector, and role, to provide context to their experiences.

*Theme 1: Cohesive networks support implementation efforts* (see Table 2 for practitioner and supervisor quotes).

**Table 2:** Practitioner and supervisor quotes related to theme 1.

Theme 1: Cohesive networks support implementation efforts	Practitioner quotes	Supervisor quotes
Sub-theme: Strong implementation context	“It's nice to be able to work with another person who has been trained in it, especially for the DSP area. Because I find that that's still so new. So that would be helpful to have more people trained.” “There's someone in my office whose role is to do DSPs. So, when I’m worried about something I might do my own ASQ but there's a parent support worker who would be able to do the DSP. So the family that I just did an ASQ with, that I am worried about, is going to be meeting with the other worker to do the DSP.” “I just kept saying to my supervisor, you have to do those webinars, you have to make sure you watch those webinars, because it's just, it's so beneficial.” “It's twofold. We've got staff that are passionate and believe in the work that they do. […] But we also have a leadership team that believes in- we’re also the lead agency for children's mental health.”	“It has been helpful though, just to explain and support staff who may have concern around the amount of time it takes, or what's the purpose to really understand what the ASQ and DSPs are.” “Now that I know that everybody's gone through the training that's there, it's become my responsibility as a manager to ensure that there's implementing of ASQ and DSP on a regular basis.” “The agency supports the ASQ and training, like so they allow time for the staff to use it and are trained to use it. […] I will prompt them if they haven't done an ASQ to say, “Can you do – you should do an ASQ on this.’ And then help them set time and figure out how to do that, because I do think it's important for these little ones. And then I’ll follow up with them, ‘Did you get that done? No? OK, did you talk to the family?’ Like those kinds questions I will prompt and really encourage them about it and show them the value of the program.”
Sub-theme: Multi-level, multi-sectoral collaborative partnerships and trusting relationships	“Right now it's virtual, but we continue to try to keep those partnerships going as best as we can to ensure that people know, we're here to be a resource and help too. And that also allows us to be able to then support the [other organization] staff. If they've got a parent that's resistant, or they're concerned, but they don't know, then they can call us. They could call me and say, hey, can you come and meet here with this parent? Or can you call this parent and we can sort of try to bridge those resources together?” “There was some confusion at first, about if we're all being trained in the same county, how are we going to know who's going to use what if we're sort of working with the same families? But that's been answered. That came about, because now there are sessions happening within each county. And having the right people around the table from within that county, and how we use those tools and support one another.”	“Trusting in the work of the partners, I think sometimes that's been a barrier as well.” “[My strengths as a leader are] to make connections between different people within the team, within the agency, within the broader community. And then to try to work through some of the problems or the barriers to collaboration and communication, to develop protocols, to make that work seamless for staff, and to have those hard conversations, those difficult conversations at a management level.” “I always feel like I’m intruding when I go over to the primary care side and, you know, chase down a parent in the waiting room. So I suppose, you know, [we need] more of a team of people, creating a team of people that we’re going to work through how to best implement this program in our organization.”

Sub-theme: Strong implementation context.

Participants expressed that successful implementation context was dependent on their organizational culture and leadership. Strong implementation context was reported as including:

(1) Clear roles and responsibilities. Participants reported that knowing *who* was doing an ASQ and DSP helped implementation efforts within and across organizations.
  Several practitioners reported feeling “confused” regarding “who does the ASQ” within their organization when multiple staff members had been trained without explicit responsibility distinction. Additionally, practitioners reported struggling with including the screening tool in their workload if they felt that developmental screening was not appropriately aligned to their role.
  Role clarity reduced confusion by minimizing the possibility of “duplicate services” (Jessica, Public Health, Practitioner) within and across organizations, defaulting screening tasks to specific people, and by providing team members with an explicit contact for ASQ and DSP support.
(2) Shared values. Participants cited that a common value of the work across the organization helped drive the implementation efforts and strategies.
  Practitioners felt that supervisors valued the use of the screening tools when they “asked us if we would be interested” (Beatrice, Education, Practitioner), “offered” the training, and “encouraged” them to complete it (Amy, Public Health, Practitioner). Both Pat (Child Welfare, Supervisor) and Yara (Primary Health Care, Supervisor) reported that a common value system amongst their staff helped to motivate team members to drive organizational-level implementation.
  Practitioners and supervisors alike felt discouraged when valuing early intervention was not shared across the agency. Participants reported “struggling” when their colleagues did not value the training, coaching, and/or early intervention practice, as colleagues who did not share these values would “put it to the back” (Sloane, Child Welfare, Supervisor).
(3) Strong leadership. Practitioners described feeling supported by supervisors that “understand the importance” of early intervention, and who were “huge proponents of early development” (Yara, Primary Health Care, Supervisor). Strong leadership was described by three participants as being supported in any way possible to ensure that practitioners were able to focus efforts on implementation, such as supporting practitioner capacity, family engagement, training, and team meetings.
  Practitioners felt unsupported by leadership when they were not encouraged to take on the training and coaching (Rebecca, ELC, Practitioner). Beatrice (Education, Practitioner) noted that training was only offered to select roles within her organization, and anyone else interested had to “personally ask” to take the training.
  Practitioners felt unable to promote ASQ and DSP implementation within their organizations when leadership was not supportive “because if they're not open to hearing, then we can say all we want” (Betty, Child Mental Health, Practitioner). Further, several practitioners felt disconnected from the implementation process due to a lack of leadership-led information dissemination. Gillian (Child Welfare, Practitioner) was not aware of organization-wide implementation because she was “not a supervisor”. Likewise, Betty (Child Mental Health, Practitioner) felt unable to drive implementation amongst her colleagues because only leadership “makes that decision.”
(4) Teamwork. Practitioners felt “comfortable and confident” (Pat, Child Welfare, Supervisor) when they had colleagues to whom they could refer to for ASQ and DSP support.
  Rebecca (ELC, Practitioner) expressed that she would validate her ASQs and DSPs with other staff members (“This is what I saw, this is what I observed, this is what I’m thinking the answer should be, but what do you guys think?”), Beatrice (Education, Practitioner) reported that other staff members had encouraged her to work as a team (“They approached me and said they would support me as much as I needed”), and Pat (Child Welfare, Supervisor) stated that her staff have team members to support ASQ use and DSP creation (“Team leads can also go with the staff, to mentor or to act as a bit of a coach”).
  However, several practitioners reported working alone when using an ASQ or creating a DSP. Working alone was reported by three participants as a consequence of insufficient organization-wide training. Yara (Primary Health Care, Supervisor) noted that working alone in her role was “just hard sometimes”.
(5) Overarching interest in early intervention. Participants that were interested in early intervention and IEMH sought out training that would fulfill their drive for knowledge. For instance, Rebecca (ELC, Practitioner) stated, “that stuff just piqued my interest… I was just like, I need to know more”, and Pat (Child Welfare, Supervisor) reported: “I know that from the completion of the ASQ and DSP, it’s also generated interest in membership in the early mental health practice leads group. It’s generated some interest in further training […] since then we've received more requests from other departments to receive the training.”
  As well, a common interest in early intervention was felt to be beneficial because practitioners and supervisors understood that they were “being trained so that we can help these families” (Rebecca, ELC, Practitioner). Participants described interest in early intervention leading to “really great ideas” (Beatrice, Education, Practitioner) and “increased attention to infant and early mental health” (Wanda, Public Health, Supervisor), as well as actual changes with behaviour, as reported by Yara (Primary Health Care, Supervisor): “the nurse practitioners have consulted me more often, have referred families to me more often.”

### 3.2. Sub-theme: multi-level, multi-sectoral collaborative partnerships and trusting relationships

Participants felt confident in organizational implementation of the ASQs and DSPs when there were established relationships between themselves, their colleagues and supervisor, their community partners, and the caregivers with whom they were working. Relationships often led to collaboration partnerships. For instance, (Betty, Child Mental Health, Practitioner) expressed feeling more confident because she knew there was a network of “people you can connect with and know that you’re a part of this together”. Building networks contributed to a “supportive understanding of each other” which allowed for practitioners to “reach out to a colleague or another agency” (Betty, Child Mental Health, Practitioner) in instances when they felt unsure in the implementation process.

Participants reported that the coaching sessions held by IEMHP were beneficial to both relationship-building within and across sectors and organizations. The coaching sessions offered the opportunity for practitioners from different regions, organizations, and sectors to come together and “hear how other people were implementing it” (Rebecca, ELC, Practitioner), as well as “know there's a support network in my community” (Betty, Child Mental Health, Practitioner).

Relationship-building in and across organizations drove implementation efforts by:

(1) increasing trust. Introducing early intervention tools to a family with whom the practitioner had a “trusting relationship” was described as “very easy” (Amy, Public Health, Practitioner). Practitioners who could “reassure moms” perceived caregivers as more trusting, reducing hesitancy towards developmentally screening (Jessica, Public Health, Practitioner). In comparison, practitioners who lacked a trusting relationship with the family were hesitant to introducing a parent-reported screening tool, describing that caregivers “may not see something that the child is able to do” (Wanda, Public Health, Supervisor), parents may inadvertently withhold important developmental information because “it’s not something most parents will tell you” (Rebecca, ELC, Practitioner), parents may purposefully withhold information due to “trust issues” (Sloane, Child Welfare, Supervisor), or parents may “decide that [they don’t] know me well enough” and refuse the screening (Beatrice, Education, Practitioner).
(2) reducing service duplication. Participants reported struggling with implementation when they felt there was no cohesive developmental screening tool policy between organizations. This struggle was described as “problematic” due to the risk of “duplication of information in different places” (Frankie, Child Welfare, Practitioner). Participants felt hesitant to introduce more information to families that accessed services from multiple organizations, reporting that parents were already feeling “tired” (Jessica, Public Health, Practitioner) and “not hearing that we’re talking the same language” (Pat, Child Welfare, Supervisor). Wanda (Public Health, Supervisor) noted that service duplication was more likely when organizations did not substantially communicate with each other. Wanda further reported that a lack of collaboration between organizations was perpetuated by “territoriality” as practitioners would often see their work as existing in isolation “rather than recognizing that everybody has a responsibility to work together on behalf of the child.”
(3) offering and receiving support. Both offering and receiving screening and practice support within and between organizations relieved worries surrounding the possibility of “incorrect” practice. Beatrice (Education, Practitioner) noted that she was “not worried” that she was going to “to mess up in some way and throw this whole family off” when practitioners in her organization would work together to review an ASQ or DSP. Betty (Child Mental Health, Practitioner) expressed that supportive partnerships reduced parent resistance to having their child screened. However, a lack of support within organizations was described as a consequence of insufficient information sharing between teams and departments by both Frankie (Child Welfare, Practitioner) and Jessica (Public Health, Practitioner).

Several practitioners noted that collaborative partnerships were often led by their supervisors. Supervisors that drove collaborative partnerships were described as assisting in developing and sustaining multi-level, multi-sectoral relationships, as well as being involved in infant and early mental health initiatives in which the teachings could be disseminated amongst staff.

“[The supervisor] is involved in a lot of different things in sort of the agency, the community, so [she] brings that back to us; and just wants to have sort of a positive work environment so [she] does try to create good relationships.” (Gillian, Child Welfare, Practitioner)

*Theme 2: Implementation success is dependent on shared perspectives* (see Table 3 for practitioner and supervisor quotes).

**Table 3:** Practitioner and supervisor quotes related to theme 2.

Theme 2: Implementation success is dependent on shared perspectives	Practitioner quotes	Supervisor quotes
Sub-theme: Individuals involved in implementation require adequate and collective awareness, knowledge, and confidence	“I would say probably the trickiest point, it's usually the family doctors, who most of them haven't been trained and if they’re aware or not, it's usually based on kind of like their private desire to increase their knowledge on the subject. Yeah, so they usually are kind of like the trickier part to explain what it is about and why do I think this child needs certain support.” “[The training] just really gave it form, it gave it some background to what I was trying to do, and it helped me to be able to manage what needed to be done within my day.” “Because we understand it better, we understand how the tools can help us in our work to help the family and have a whole clearer understanding of the resources that go along with it. And how to code it and what that means… that, I think, that has certainly made a difference.”	“Because we have different representatives from different departments within the organization, it allows for them to bring information back to their individual teams, which is inclusive of this training. And I ensured as we looked at which group should be trained, we had our children in care, we had our family support, we included some of our children/youth mental health staff.” “We have a really excellent infant mental health practice leads program in our agency. I think what it's done is that it's expanded the knowledge to the teams like family support team and child and care worker teams that they might not have thought about infant mental health before. So I think it's just – it's grown the knowledge for sure and when I bring up infant mental health like, you know, what's this baby telling us, what do we see, they’re thinking now that babies do have mental health so I think that's really promoted it; like there's a great knowledge.”
Sub-theme: Consistent and critical conversations on implementation strategies and follow-up	“I'm the chair of our team, we talk about ASQ's and DSPs at every meeting just as a standing agenda item so it's always kind of coming up […] and then [the manager and director] support our communication if we have questions, our manager supports that conversation with [Facilitator].” “Team meetings are generally to be honest administrative, we don't have team meetings where we’re talking about, very rarely, like it has happened but where we’re talking about clinical work or stuff like that, that's not what team meetings are for. It's kind of sad actually; like we don't do any type of group work like that.” “The protocol is the teacher and the ECE, early childhood educator, they communicate to one another, they see a need, they communicate that to the Special Education Teacher and the Special Education teacher communicates with them and they figure out who they need to bring in from the board office or what needs to be administered.”	“Early intervention and prevention is certainly one of our pillars and one of our strategic plans, as indicated in our organizational operational planning. With that comes conversations around prevention and early intervention, which is inclusive of early mental health. And so at a leadership level, in conversations with each other, we're also within capacity to have those conversations with each other to support continued learning. And to consider how we might formalize the use of ASQ and practice across those departments that have been trained.” “I think the coaching sessions really helped when things are so busy to help staff be reminded about not only the conversations, but the purpose and how it can help a child.”

### 3.3. Sub-theme: individuals involved in implementation require adequate and collective awareness, knowledge, and confidence

Participants expressed how implementation of ASQs and DSPs was facilitated by the network of professionals having a common level of awareness, knowledge, and confidence in early intervention practice. These shared characteristics were described as created and sustained through several methods:

(1) Training. The standardized training allowed for all participants, regardless of sector or position, to learn the same information. However, when supervisors and practitioners did not undertake the same, or any, training, participants described obstacles to implementation. Beatrice (Education, Practitioner), Gillian (Child Welfare, Practitioner), and Wanda (Public Health, Supervisor) noted that untrained service providers lacked necessary understanding of early identification and early intervention practice to drive implementation efforts. Amy (Public Health, Practitioner) described collaborating with untrained coworkers and community partners as “a work in progress”.
  Practitioners were reported to be less likely to complete IEHMP training because “it seems onerous” (Frankie, Child Welfare, Practitioner), because “it might be a little bit overwhelming” (Amy, Public Health, Practitioner), and because they “didn’t think it was super relevant to [their] job title” (Gillian, Child Welfare, Practitioner).
(2) Coaching. If participants felt they did not fully understand the learned topics following training completion, they reflected that the coaching sessions were opportunities to standardize their understanding. Betty (Child Mental Health, Practitioner) noted that “it just allowed me to be able to practice it, see what other, and hear what other, people have been using it in their agencies”. Jessica (Public Health, Practitioner) expressed that learning from other experienced professionals was “really helpful”. Sloane (Child Welfare, Supervisor) saw the coaching sessions as opportunities to listen to the struggles of “different people from around the province that took the training” to plan for solutions in the event that her team was struggling with comparable issues.
  Following coaching sessions, several participants reported increased feelings of confidence in early intervention practice, screening skills, and family engagement skills. Sloane (Child Welfare, Supervisor) reported that, prior to coaching, a lack of confidence in screening skills had been a setback for her organization because “people get stumped on and don’t want to do is the scoring.”
  Practitioners particularly felt confident engaging families if caregivers had a shared awareness and knowledge of early intervention practice. While some practitioners “didn’t experience any challenges” engaging families who were familiar with the ASQs (Betty, Child Mental Health, Practitioner), other participants described the use of the ASQs and DSPs as “not well-understood or accepted” by caregivers unfamiliar with the ASQs (Gillian, Child Welfare, Practitioner). Several participants felt that a lack of parental awareness and knowledge had the potential to negatively impact their ability to implement the ASQ with particular families. Betty (Child Mental Health, Practitioner) stated:
  “I think the biggest piece is that we can do all this training and all of the ASQs… if we don’t have buy-in from the parents, then it doesn’t make any difference. We need to somehow educate the families as well.”
  Sloane (Child Welfare, Supervisor) also asserted that “we have to educate them”, indicating that caregiver awareness and knowledge on the use of the ASQs and DSPs would reduce parental reluctance.
  Educating caregivers on early intervention practice was cited as allowing both parties to move forward with a shared level of understanding: “When people know about the ASQ, it’s easier to implement” (Amy, Public Health, Practitioner).
(3) Facilitator engagement. Practitioners felt aware and knowledgeable following continuous communication with the Facilitator (the IEMHP staff member conducting the coaching sessions). Betty (Child Mental Health, Practitioner) reported that communicating with the Facilitator in the coaching sessions gave her “that opportunity to really feel I understood it, and how to implement it before I went off to try it.” The support from the Facilitator was highly valued by the participants, who reported that “knowing I can shoot an e-mail if I’m not sure and have a personal response” helped build and maintain their confidence with the implementation of the ASQs and DSPs (Amy, Public Health, Practitioner).

### 3.4. Sub-theme: consistent and critical conversations on implementation strategies and follow-up

When participants were confident in their understanding of infant and early mental health and early intervention practice, they would begin to increasingly include these topics in conversations with colleagues, parents, and other professionals in the field. These conversations helped those participating to develop and maintain shared awareness, knowledge, interest, and confidence.

Discussions focused on implementation of developmental screening tools and early intervention practice were championed by:

(1) A dedicated IEMH group. Several organizations had “IEMH Groups”: an internal group focused on “ongoing conversations” and disseminating information. Staff members from various departments would attend the groups and “take that information back to our team” (Frankie, Child Welfare, Practitioner). For example, Pat (Child Welfare, Supervisor) discussed how the IEMH group at her organization included practitioners from multiple departments, allowing for the knowledge gained from the training to be disseminated throughout the organization. Knowledge dissemination in this fashion was viewed as important because other departments “might not have thought about infant mental health before” (Sloane, Child Welfare, Supervisor).
(2) Regular meetings including IEMH and implementation topics. Sloane (Child Welfare, Supervisor) described that regular inter-departmental meetings “made staff aware about infant mental health” and were helpful to practitioners who required guidance with implementation. Wanda (Public Health, Supervisor) reported that regular team meetings were a space for her staff to assist each other by offering advice or sharing lived experience.
  Frankie (Child Welfare, Practitioner) reported that her team would meet “on a monthly basis” to “talk about ways that we can encourage interventions”. Similarly, Sloane (Child Welfare, Supervisor) noted that her team's monthly meetings were used to “go over the ASQ together” and work as a group to “rectify” any “problems” with the implementation of the ASQs and DSPs. Jessica (Public Health, practitioner) spoke about how meeting “every six weeks or so” to discuss the ASQs and DSPs promoted her organization's “strong focus” on early intervention.
  However, several organizations did not incorporate IEMH, the use of early intervention tools, or implementation strategies into their intra- or inter-departmental meetings. In some instances, including a focus on implementation strategies and follow-up in team meetings was either scarce or excluded entirely (Yara, Primary Health Care, Supervisor).
(3) Communication with trained colleagues and providers. When participants were in spaces that included trained practitioners, many felt emboldened to discuss the information they had gathered, listen to the experiences of others, and ask for clarification.
  Gillian (Child Welfare, Practitioner) reported that she would collaborate with a trained professional in a different role from her own to discuss and learn from each other's experiences. Rebecca (ELC, Practitioner) noted that two staff members who carpooled to and from work would “share information back and forth” to remain informed with the organization's implementation process. Amy (Public Health, Practitioner) similarly reported “sharing” and “brainstorming” with trained colleagues when she was confused with the next steps in the implementation strategy.
  When communication was poor or scarce, participants experienced feelings of frustration. Jessica (Public Health, Practitioner) felt hindered in her organization's implementation efforts due to “challenges” of “connecting” with other trained professionals. Wanda (Public Health, Supervisor) described insufficient communication as a “barrier” to service coordination that would streamline implementation efforts.

*Theme 3: Established organizational policies increase implementation opportunities* (see Table 4 for practitioner and supervisor quotes).

**Table 4:** Practitioner and supervisor quotes related to theme 3.

Theme 3: Established organizational policies increase implementation opportunities	Practitioner quotes	Supervisor quotes
Clear protocols and procedures incorporating screening tool use	“I think if it was more common practice then we would be using it more commonly and maybe talking about it more commonly.” “I feel if I had more time then I would do it more often.” “I don't think it's helpful when it's not part of our Ministry-standardized system, then it feels kind of like this thing on the side. That's when people get confused about ‘when, who, how’ should it be used.”	“For each department, we're in the midst of formalizing our procedure development, so that the way in which a case is managed is consistent, department by department.” “There was just concern around the time in which the completion of an ASQ and then maybe even perhaps a DSP would take away from current responsibilities and obligations within their workload.”
Accessibility to early intervention information, tools, and best practice guidelines	“Obviously, community resources are limited and everywhere is a waiting list, but it definitely gives us an opportunity to refer kids sooner and put them on these waiting lists sooner than later and maybe hopefully they will get an actual professional service before it's too late or before it's requiring major interventions.” “I can tell you for our agency, where we have it stored. I have to log into what we call our VPN, so I have to log into that, then I have to click another thing, to get through another password, then I have to go into a separate drive, in our system, and I have to scroll and find it, and just to be honest – that is, I think, a barrier.”	“Once they’re done with the nurse practitioner I take them downstairs. I have a program room down there where I can quickly throw out a baby mat and some toys and comfortable chairs. It's a large room. Everyone can spread out. You know, parents usually feel more comfortable and the child can roam and play and be occupied.” “The other problem is that the ASQ isn't electronic and so that's really problematic because we might be able to get more referrals or information for the electronic medical record. It has to be electronic. And working remotely or, you know, virtual service delivery you need electronic tools. So that's been – that's a big barrier I would say.”

### 3.5. Sub-theme: clear protocols and procedures incorporating screening tool use

Practitioners felt inclined to use the ASQs and create the DSPs when these tasks were explicitly incorporated into their daily workload. Several factors needed to be considered when implementation of the ASQs and DSPs was meant to be routine practice:

(1) Staff capacity. All practitioners felt that their capacity to modify their workloads to accommodate the ASQs and the DSPs was bolstered when there was adequate time and staff available. Supervisors were cited as enabling this capacity building by advocating for their staff (Jessica, Public Health, Practitioner), ensuring “there’s enough staff to cover” (Rebecca, ELC, Practitioner), and allocating resources to ensure practitioners can freely “take the time” to be trained, engage families, practice screening skills, use the ASQs, and create the DSPs (Amy, Public Health, Practitioner).
  However, several participants struggled to complete training, regularly use the ASQs, and create DSPs when their regular workload had not been accommodated to include additional tasks. Practitioners expressed that implementation efforts suffered because the “job is already full as it is” (Gillian, Child Welfare, Practitioner), staff are “booked so solid” (Yara, Primary Health Care, Supervisor), and staff were instructed to “take it on top of your regular workload” (Frankie, Child Welfare, Practitioner). Yara (Primary Health Care, Supervisor).
(2) Organizational expectations. Implementation efforts were driven when practitioners were expected to complete the training and coaching and implement the ASQs and DSPs. Amy (Public Health, Practitioner) described the implementation of the ASQs as “a part of the expectation that this is what the nurses will do since we got the official training”. Sloane (Child Welfare, Supervisor) had encouraged her entire team to be trained, describing an “expectation” for practitioners to “do the ASQ or at least offer it to the family”. This expectation was reinforced by supervisors who viewed the training and coaching as essential to policy and professional development. Pat (Child Welfare, Supervisor) described “ongoing expectations” of her team to attend coaching sessions to improve organizational screening procedures. Similarly, Wanda (Public Health), expected her staff to complete the training and coaching as a method of establishing organization-wide best practice.
(3) Organizational policy. Practitioners who were mandated to use ASQs were persistent in their implementation efforts. Persistent implementation efforts were driven by previously established organizational guidelines (Jessica, Public Health, Practitioner), long-standing organizational screening tool use and early intervention practice (Betty, Child Mental Health, Practitioner), and the need to meet ministry standards (Amy, Public Health, Practitioner).
  When the ASQ and DSPs were not embedded in organizational policies, practitioners would focus attention on first completing tasks that were mandated, as work “goes in priority” (Beatrice, Education, Practitioner). ASQs and DSPs were not considered high priority when alternative early intervention practice had been a part of the staff's routine tasks for a long time (Gillian, Child Welfare, Practitioner), other early intervention practice was ministry-required (Frankie, Child Welfare, Practitioner), or other job requirements were considered more important (Gillian, Child Welfare, Practitioner).

### 3.6. Sub-theme: accessibility to early intervention information, tools, and best practice guidelines

Access to early intervention information, tools, and best practice guidelines was essential for participants to successfully implement the ASQs and DSPs. Accessibility was improved through:

(1) Technology. Several participants stressed the importance of technology as a way of streamlining implementation efforts. The use of technology, such as “virtual and phone” (Betty, Child Mental Health, Practitioner), facilitated implementation by reducing scheduling and transportation barriers. Working with families “over the phone” was described as “easier” by both Yara (Primary Health Care, Supervisor) and Gillian (Child Welfare, Practitioner). Both practitioners felt they were better able to accommodate the caregiver's needs when they offered phone screenings, specifically referring to comfort (Yara, Primary Health Care, Supervisor) and scheduling (Gillian, Child Welfare, Practitioner).
  When technology was inaccessible, in-person sessions were described as a “barrier” due to possible difficulties of transporting young children to the organization (Betty, Child Mental Health, Practitioner), and scheduling conflicts (Gillian (Child Welfare, Practitioner).
  Technology was also considered a useful tool to accessing early intervention information and best practice guidelines. Rebecca (ELC, Practitioner) reported that she “did every one” of the training modules because she was able to access them from her home. Gillian (Child Welfare, Practitioner) had received partial training in-person but acknowledged that the virtual training positively influenced her screening tool use: “I can say that I really didn’t do many ASQs until I had the more recent virtual training”.
  Two practitioners felt challenged when there was a lack of organizational technology use, including lack of an electronic ASQ available on a Canadian server, as the current virtual ASQ houses all data in the United States. Accessing the paper version of the ASQ was reported as not “user-friendly” and “problematic” (Wanda, Public Health, Supervisor). Frankie (Child Welfare, Practitioner) expressed that it was “very challenging” and “discouraging” to access the paper version of the ASQ, reducing her motivation to implement the tool within her organization.
  Wanda (Public Health, Supervisor) reported that an electronic ASQ would simplify the referral and information-sharing processes between organizations. Wanda also emphasized that “you need electronic tools” when practitioners were working remotely or offering virtual services, as the inconvenience of using paper tools was felt to outweigh the risk of using no screening tool at all.
(2) Administration space. Yara (Primary Health Care, Supervisor) found that working in a location with a dedicated space to screen children improved her capability to “incorporate the ASQs”. Several practitioners reported that inaccessibility to an appropriate space to conduct ASQs and create DSPs negatively impacted their implementation efforts. Gillian (Child Welfare, Practitioner) described an “issue” with working in a physically small organization, due to a lack of dedicated screening administration areas. Two practitioners conducted ASQs when visiting family homes, but described these circumstances as “distracting” due to a lack of privacy. Similarly, Frankie (Child Welfare, Practitioner) would attempt to conduct ASQs virtually by using a scanned version of the ASQ at family residences but often struggled with the lack of suitable space: “There’s not a surface to set your computer on to be typing”.
(3) Funding. Participants reported obstacles to implementation when financial resources were limited. Adequate funding was necessary to designate enough staff to the responsibilities within the organization to ensure the ASQs and DSPs were able to be completed. Wanda (Public Health, Supervisor) reported that the cost of staff to use the ASQs with families at their residences included “mileage and time”, increasing organizational expenses, and lowering leadership motivation to send staff out on these home screening visits.
  “Insufficient funds” was further reported as a problem for resourcing required to minimize or eliminate waitlists (Wanda, Public Health Supervisor). Waitlists are a significant issue when attempting to implement development screening tools for children, as children may age out of the developmentally-appropriate screening age range.
  Families also faced access barriers to receiving developmental services when the developmental screening tools were beyond parental literacy levels, or not provided in the caregiver's native language. Despite multiple published translations of the ASQ, each language version is sold separately and, thus, may not be financially feasible for the organization to purchase.
(4) Parental literacy and language. Rebecca (ELC, Practitioner) noted that she could not assume that all caregivers are capable of reading at the 4- to 6th- grade level in which the ASQs are written. She expressed that low parental literacy “makes it a little harder” to use the ASQ as a parent-completed tool. Jessica (Public Health, Practitioner) reported that she “struggles” when she is working with “a family that doesn’t speak English”, as her organization does not have access to other-language ASQs. Jessica would attempt to overcome these barriers by utilizing a translation service over the phone but noted that this process was “really difficult” due to the cumbersome nature of speaking through a “language line” to work through the ASQs and DSP.

*Theme 4: COVID-19 guidelines create organizational challenges* (see Table 5 for practitioner and supervisor quotes).

**Table 5:** Practitioner and supervisor quotes related to theme 4.

Theme 4: COVID-19 guidelines create organizational challenges	Practitioner quotes	Supervisor quotes
	“Not only are we dealing with the children, but we’re all trying to do the Covid cleaning too, and the Covid paperwork and then dealing with the children and working on ASQs and getting all of the other documentation that needs to be done, there's just, there's not enough hours when you’re at work to do it all.” “The other approach too when we weren't doing in home visits during COVID, when things were shut down is again we would mail it out to the mom and then we’d be able to do it with them over the phone. So they have their copy in front of them and that helped too but again just, it was really long, a long process of – and then with mom being on the phone the whole time, it was a challenge to complete them.”	“We moved into the virtual so they would start to do it virtually. It's really hard to do it virtually. There's too many distractions for the parent and etcetera, etcetera. Much better to do all of those assessment tools, anything that's a tool really with a person in their home or a setting that's similar to home.” “The biggest I think barrier, and this is across the board throughout our practice for the last almost closing in on 2 years here is while learning, even my staff who are sitting on the same team, who typically be sitting four feet away from each other while doing such training, they can converse with themselves as they're doing the training. To bounce ideas are talking about a common client that they're both familiar with, to kind of to add more context, which they were unable to do.”

The global coronavirus disease (COVID-19) pandemic impacted working conditions for early childhood practitioners and supervisors.

(1) Reduced opportunity for in-person screening. Several practitioners preferred to complete an ASQ with the parent and the child present, as “observing the child” was felt to be crucial to ensuring an accurate score on the screening tool and “face-to-face fosters more relationship building” (Yara, Primary Health Care, Supervisor). Jessica (Public Health, Practitioner) reported struggling when in-home visits were indefinitely restricted, stating that “it was a challenge to complete them” when her organization shifted away from in-person service delivery. Wanda (Public Health, Supervisor) reported that her staff felt that it was “really hard to do it virtually”, describing “too many distractions for the parents” when practitioners were not sitting with them face-to-face to complete the tool.
(2) Shifting roles and responsibilities. Participants experienced role and responsibility changes within their organizations, and/or partner organizations, in response to the global pandemic. Betty (Child Mental Health, Practitioner) and Yara (Primary Health Care, Supervisor) felt disheartened by the shifted roles in their partner organizations, both reporting that COVID-19 regulations shut down organizations to which they would have referred families: “Why would we refer our struggling families to somewhere that isn’t even operating right now?” (Yara, Primary Health Care, Supervisor)
(3) COVID-19 guidelines reduced communication. Several practitioners reported that they were connecting less frequently with internal colleagues and external community partners. Betty (Child Mental Health, Practitioner) used to collaborate in-person with Early On centres to engage families and conduct early intervention practice but was unable to continue this partnership during the global pandemic. Two participants reported that colleagues no longer working in a shared space due to COVID-19 regulations negatively impacted cooperation on implementation efforts. Sloane (Child Welfare, Supervisor) expressed that she felt her team members would be more supportive if they were “in the office like they used to be, in one room together”, reporting that “if we weren’t in COVID there might be more collaborative work”. Pat (Child Welfare, Supervisor) described her team working in separate areas as “the biggest barrier”, as colleagues were no longer able to “bounce ideas” off each other, nor were they able to “converse with themselves as they’re doing the training”.
(4) Disrupted training. IEMHP's training and coaching was offered in-person pre-pandemic and shifted to a virtual model during the pandemic. The interruption in training caused a practitioner to start over from the beginning, increasing total time spent on the model (Gillian, Child Welfare, Practitioner). Other practitioners were unable to begin or complete the training and coaching model because they were re-deployed to respond to COVID-19 needs: “the only reason that a Public Health Nurse isn’t trained is because they’re still in COVID response” (Wanda, Public Health, Supervisor). Wanda unsuccessfully advocated for re-deployed staff members to be involved in the training and coaching: “I have somebody’s who’s deployed right now who I tried to get back for this last training and they weren’t released” (Wanda, Public Health, Supervisor).
  Other practitioners felt their learning process throughout the virtual training and coaching was disrupted due to factors outside of their control, such as sound issues, poor internet connectivity, and struggles “learning from home” (Beatrice (Education, Practitioner).

## 4. Discussion

Implementation is a complex, multi-dimensional process that intentionally translates knowledge gained from research into practice (47). Evidence-based interventions are created and utilized in community-based organizations to produce observable and measurable change (48). However, research outputs often face challenges mobilizing into practical application at the population-level (49, 50), community-level, or organization-level (51). It is estimated that nearly two-thirds of attempts to organizationally implement novel practice fail (52). Through semi-structured interviews with practitioners and supervisors who work directly with children under the age of 6, this study characterized current barriers and facilitators to implementing the Ages and Stages Questionnaires and Developmental Support Plans at the organizational level following completion of a training and coaching model.

The first theme presented in this paper supports prior research that indicates implementation of new knowledge into health services practice is positively influenced by organizational context that is inclusive of organizational culture and leadership (53). The findings of this study further align with Paynter et al. (54) results that indicate organizational culture is directly related to increased evidence-based practice, as well as Glisson's (55) work that suggests organizational context and culture are linked to service adoption and delivery. This study adds to the current literature by identifying specific elements of organizational culture and leadership that influence the implementation efforts of supervisors and practitioners working with children under the age of 6. This study also adds to the current literature on implementation of developmental screening tools and early intervention by including supervisor perspectives, as research in this area has largely focused on provider and parent experiences (56–59). In this study, practitioners and supervisors alike felt supported in their implementation efforts when there was a cohesive network of trained professionals and leaders that valued the use of developmental screening tools and early intervention practice, had explicitly defined roles and responsibilities, were interested in early intervention practice, and with whom they could work as a team to use and review the developmental screening tools and developmental support plans. All the factors listed above were reported to be influenced by sufficient information sharing between teams and departments.

Previous research has also highlighted the importance of parent-provider relationships in driving implementation of early intervention (60, 61). While relationship-building and community teamwork have been found to positively contribute to implementation effectiveness (62, 63), this study emphasizes the importance of multi-level, multi-sectoral partnerships that, while including the parent-provider relationship, also detail the necessity for trusting relationships between practitioners, supervisors, and external community partners. This study expands on past work by outlining key factors to creating and sustaining intra- and inter-organizational collaborative partnerships. In particular, this study highlights that increasing trust, reducing service duplication, and both offering and receiving support with developmental screening tasks and implementation strategies are critical pieces to positive relationship-building in and across organizations.

Further, prior research notes that leadership support is essential to developmental screening and early intervention implementation success (53, 64). This study elaborates on this concept by summarizing the behaviours of a supportive leader that drive implementation, such as building relationships intra- and inter-organizationally, encouraging and promoting professional development through training and coaching, supporting staff capacity to incorporate developmental screening and early intervention into routine practice, and ensuring adequate funding.

The second theme presented in this study addresses the importance of shared perspectives between parents, practitioners, and supervisors in developmental screening and early intervention implementation. While shared perspectives and attitudes have been noted as influencing successful implementation of other health services innovations (65, 66), this study's focus on multi-level, multi-sectoral shared perspectives with regards to developmental screening and early intervention helps to fill a gap in early intervention implementation literature. Shared perspectives, in the context of this paper, are inclusive of collective awareness, knowledge, and confidence. A lack of collective knowledge, as reported in this theme, is supported by past work that indicates lack of provider and parental knowledge are barriers to implementation of developmental screening (59, 67). This study further identifies that, in addition to inadequate practitioner knowledge, a lack of supervisor/leadership knowledge negatively influences the uptake of developmental screening by practitioners, as organizational training is often dependent on supervisor understanding and support.

The second theme also supports previous research that has reported a lack of training (56, 68, 69), and a lack of information regarding screening tools (70), as barriers to adequate capacity to support developmental screening implementation. This study adds to the current literature by providing an account of facilitators that may mitigate barriers caused by insufficient training and knowledge. First, practitioner and supervisor implementation efforts were facilitated when the implementation process, developmental screening tools, and early intervention were a focus of regular conversation within teams, across departments, and between community partners. Regular conversations dedicated to developmental screening, infant and early mental health, and implementation facilitated knowledge translation and dissemination, helping to build a common level of awareness, knowledge, and confidence. Regular conversations were facilitated by coaching sessions, dedicated organizational infant and early mental health groups, and routine inter-departmental meetings that explicitly incorporate discussions on developmental screening tool use and implementation strategies. This finding is supported by Simpson and Dansereau (71) study that suggests established, high quality discussions, as well as informal, clear communications, positively affect implementation effectiveness.

Confidence also plays an important role as a facilitator in implementation efforts. Previous work identified that a practitioner's lack of confidence in developmental screening skills is a barrier to implementation (72, 73). In this study, practitioners reported feeling discouraged implementing the ASQs and DSPs when they, and/or their colleagues, were not confident in their screening and parent engagement skills. This study illuminates three distinct facilitators to increase confidence to bolster implementation efforts: coaching sessions, implementation support from a facilitator, and caregiver knowledge on developmental screening and early intervention. Therefore, confidence to drive developmental screening and early intervention implementation may be strengthened by collective practitioner and caregiver knowledge. This collective knowledge can be supported through educational opportunities, such as coaching, that are openly offered to practitioners and caregivers alike, or practitioner-led knowledge dissemination to caregivers. Suggestions for practitioner-led knowledge dissemination strategies include handing out pamphlets when meeting families, distributing a webinar to families by e-mail, or discussing developmental screening and early intervention at childcare pick-up or drop-off.

The third theme presented in this paper suggests that a lack of established organization policies negatively impacts implementation of developmental screening tools and early intervention. Previous work has documented that insufficient time, staff, and physical environments, as well as competing priorities, are barriers to routine screening (57, 66, 74). This study expands on this knowledge by evaluating practitioner experiences when workloads were not modified to accommodate additional tasks, and by identifying possible mitigating factors to overcome the aforementioned barriers. Practitioners experienced frustration and discouragement with the incorporation of the developmental screening and early intervention practice into their daily workload when no, or few, accommodations were made to account for the time, physical space, staff, and resources necessary to complete the ASQs and DSPs. Practitioners also felt less motivated to use the ASQs and create the DSPs when access to the tools were cumbersome due to insufficient technology, resources, or funding. However, practitioners felt compelled to incorporate the ASQs and DSPs into their practice when the use of the tools was organizationally expected, and ministry mandated. While previous research (65) suggests providers are more susceptible to new practice inclusion when there are specific written organizational policies, this study underscores that explicit leadership-led expectations of routine practice, as well as accessibility to essential information and resources, are concurrently critical to successful organization-level implementation.

Finally, the fourth theme presented in this paper focuses on the role of the COVID-19 global pandemic as a barrier to developmental screening and early intervention implementation. The COVID-19 global pandemic impacted implementation efforts by introducing regulations intended to slow down transmission of the virus, such as virtually converting, or indefinitely suspending, in-person services. Remote service delivery decreased communication between colleagues, diminished collaboration opportunities between organizations, and reduced ease of ASQ use with families. The COVID-19 regulations also shifted practitioner roles and responsibilities, deploying staff who would otherwise have been trained and coached on the ASQs and DSP, thus reducing the number of staff who could have implemented the tools.

The theme of COVID-19 creating organizational challenges is consistent with additional studies that have found practitioners struggled with the transition from in-person to virtual or phone screening (75). Additional barriers to implementation of evidence-based practice during the pandemic included lack of privacy, technological obstacles, and preference for in-person services (76). Literature on the impact of COVID-19 on implementation of developmental screening tools and early intervention is currently limited. To the author's knowledge, this study is the first to contribute to the growing body of developmental screening and early intervention implementation literature by providing accounts of practitioner and supervisor experiences of training, coaching, and implementation during a global pandemic.

### 4.1. Key implications

This qualitative study can inform the implementation efforts, strategies, and process of practitioners and supervisors providing developmental screening and early intervention to children under the age of 6. The thematic analysis presented in this paper can be used to inform future framework development for organization-level implementation of developmental screening tools and early intervention. Prior to formal framework development, individuals seeking to successfully implement developmental screening tools within their organizations may utilize the themes and sub-themes presented in this paper as a guide to proactively address barriers and promote facilitators by identifying their organization's readiness status for implementation.

For example, prior to implementation, those involved in the implementation process should identify whether their organizational culture is supportive of implementation (e.g., is there a strong leader? Is there role clarity? Are values shared across the organization?), as well as whether there are collaborative partnerships available to leverage (e.g., are there any other staff members that could help administer the tools? Do staff, supervisors, and caregivers share levels of knowledge and confidence?), whether there are opportunities for consistent implementation conversations (e.g., is there time during weekly meetings to add implementation strategies to the agenda?), and whether policies or procedures must be developed to outline the routine use of developmental screening tools and early intervention services (e.g., is there a designated administration space in the organization?).

Identifying the organization's readiness status for implementation in relation to this paper's themes and sub-themes will help to identify areas that require development or strengthening prior to implementation. Once areas of need have been identified, strategies to meet needs should be undertaken.

For instance, if an organization has identified that practitioners and caregivers do not share the same level of knowledge regarding developmental screening tools and early intervention, they may strategize to build shared knowledge through several methods. First, one service provider and one supervisor from each department within the organization may complete the training and coaching model. The service providers and supervisors can then bring the learned information to team meetings and become internal learning facilitators. Second, caregiver knowledge could be strengthened through dissemination of educational resources such as videos or brochures, or organizations may invite caregivers to participate in information sessions where practitioners share what they learned in the developmental screening and early intervention training and coaching sessions. As well, if an organization has identified they do not have sufficient collaborative partnerships, leadership may seek out potential partners within their community that have a shared vision for improving developmental outcomes and establish formal memorandums of understanding to clarify roles and responsibilities, ensure accountability, and facilitate the sharing of resources and information.

Organizations that utilize the themes and sub-themes presented in this paper may find that they experience a more seamless implementation process, as they will be able to recognize and respond to organizational-level implementation gaps before beginning the implementation process. Future work should include a focus on formalizing these themes, sub-themes, questions, and strategies into an organizational-level implementation framework.

### 4.2. Strengths and limitations

This study helps to fill a gap in current understanding of the challenges faced by early childhood service providers and supervisors when attempting to implement developmental screening and early intervention services in their organizations. Organizations who wish to implement developmental screening and early intervention in practice may use the themes and sub-themes presented in this paper as a guide to recognize and address the barriers they may face, and the facilitators they must promote, prior to, and throughout, the implementation process.

Further, to the author's knowledge, this is the first study identifying barriers and facilitators to implementing developmental screening tools and early intervention in organizations working with children under the age of 6, following training and coaching that has occurred during a global pandemic. As such, the barriers and facilitators described provide a useful starting point for implementation of early intervention practice during typical practice, as well as possible future pandemics.

Finally, this paper is the first known research to identify barriers and facilitators to developmental screening following training and coaching in the Canadian context. The authors believe that the understanding gained from this study is not unique to the Canadian context and can be applied to any organization in countries that have similar childcare organization infrastructure and resources. Thus, this paper can provide guidance to supervisors and practitioners intending to implement early intervention practice with families within their organizations.

There are also several notable limitations to this study. First, the semi-structured interviews were held with practitioners and supervisors who work in Canada. As such, it cannot be assumed that the barriers and facilitators highlighted by these discussions can be generalizable to countries that are infrastructurally dissimilar to Canada, as early intervention practice and guidelines vary across countries. Further, the sample size in this study (7 practitioners, 4 supervisors) may not be representative of all perspectives held within and across organizations that have completed IEMHP's training and coaching model. A larger sample that consists of an equal number of practitioner and supervisor perspectives across all sectors involved in IEMHP's training and coaching model may provide deeper insight to the barriers and facilitators faced. Further, primary caregiver characteristics were included as both barriers and facilitators identified by practitioners and supervisors; however, primary caregivers were not included in the participant sample. As such, direct insight into the barriers and facilitators that primary caregivers experience was not included in this study. Future research should expand the participant sample to include primary caregivers to broaden the general understanding of the barriers and facilitators to implementation of developmental screening and early intervention. Moreover, while several sectors were included in this analysis, themes were not isolated to specific sectors, and the barriers and facilitators to implementation may, in some cases, be sector-specific (e.g., parent reluctance was commonly discussed with participants working in the Child Welfare sector, whereas this topic was not as salient with the other sectors). Future research should focus on identifying sector-specific barriers and facilitators to early intervention practice implementation, utilizing a larger sample size across all Canadian provinces.

### 4.3. Reflexivity statement

The researchers involved in this project have attempted to present each practitioner and supervisor's narrative appropriately, and to the fullest extent possible as outsiders to their work. The first author (KP-K) is a doctoral candidate whose project focuses on the implementation of developmental screening tools in organizations that work with children under the age of 6, in partnership with the Infant and Early Mental Health Promotion group that created and runs the training and coaching the participants completed. The first author has no first-hand experience introducing and using ASQs and DSPs with families. The secondary coder (CP) is a doctoral student studying rehabilitation science and does not have first-hand experience using developmental screening tools or early intervention practice. The analysis presented in this paper has been co-produced by the first and second authors' interpretations and the practitioners and supervisors who generously gave their time, insight, and feedback to offer and validate the perspectives on barriers and facilitators to implementation.

### 4.4. Conclusion

Training and coaching supervisors and practitioners to administer developmental screening tools and create developmental support plans can positively impact the developmental trajectories of the children with whom they engage in early intervention. Organization-level barriers and facilitators to developmental screening administration and developmental support plan creation should be recognized and addressed prior to, and throughout, the implementation process. Additional research is needed to identify individual-level and systems-level strategies to reduce barriers and promote facilitators. Future quantitative research is also necessary to determine if strategies to reduce implementation barriers and promote implementation facilitators influences the number of practitioners administering the ASQs, the number of children screened, and the number of families utilizing early intervention services.

## Data Availability

The raw data supporting the conclusions of this article will be made available by the authors, without undue reservation upon request to the corresponding author.
